# Supplementary material for: Prohibitin 2 is Involved in Parkin-Mediated Mitophagy in Urothelial Cells of Cattle Infected with Bovine Papillomavirus
Source: Pathogens. 2020 Jul 29;9(8):621. doi: 10.3390/pathogens9080621 (PMC7460215; doi:10.3390/pathogens9080621)
Supplement: Supplementary file 1 [file pathogens-09-00621-s001.pdf]

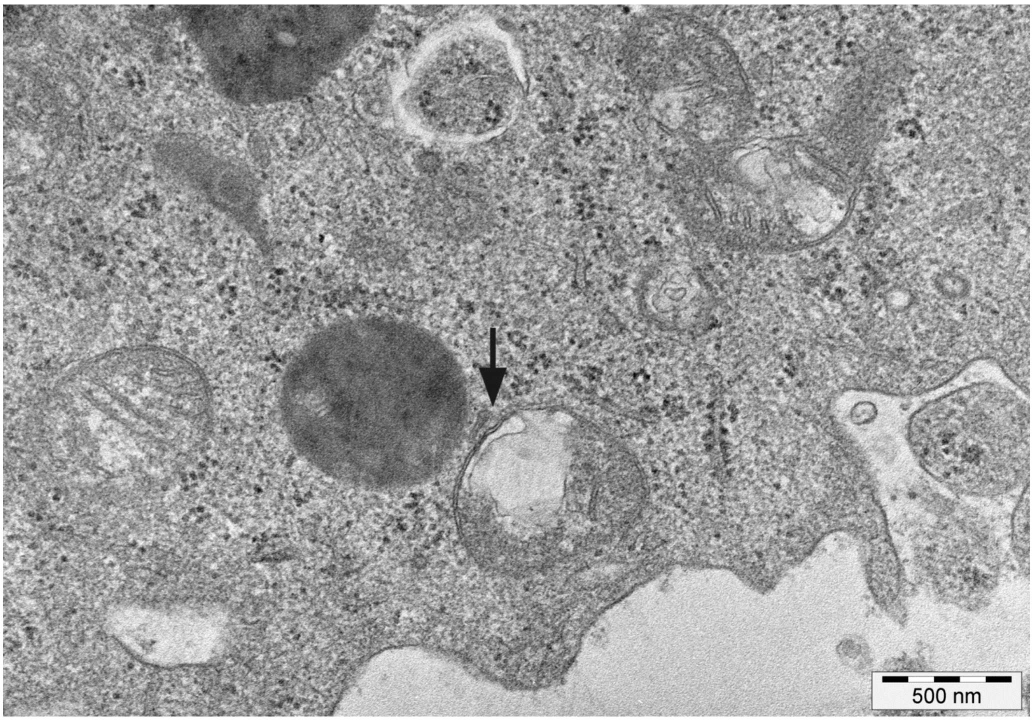

Supplemental Figure S1. Transmission electron microscopy image of a perturbed crista, characterised by an increased width of both its junction and lumen, is shown. Isolation membranes (also called phagophores) are localised on the opposing face of the damaged OMM (arrow).

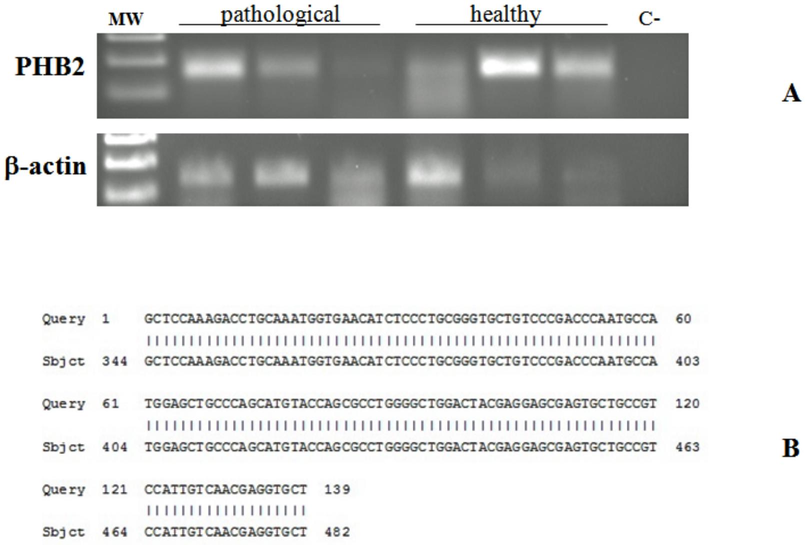

Supplemental Figure S2. *PHB2* cDNA amplification by PCR in normal and neoplastic bovine bladders as compared to actin. Lane 1: molecular weight marker (DNA marker ladder); lanes 2–4: healthy bladder

samples; lanes 5–7: three representative papillary urothelial cancers; in the last channel: negative control (RNA without reverse transcriptase subjected to PCR analysis). The lower part of the figure shows the alignment of the sequences, which revealed 100% identity with bovine *PHB2* transcript present in GenBank (*Bos taurus* prohibitin 2 (PHB2), mRNA: NM_001046198.1).

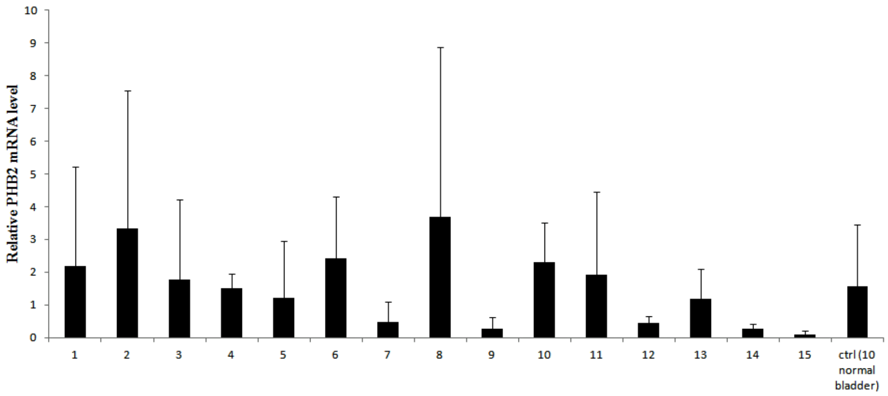

Supplemental Figure S3. Real time RT-PCR. *PHB2* mRNA levels in the 10 normal control (CTRL) and in the 15 neoplastic bladder samples. Data are expressed as mean ± S.E.M. of three separate experiments performed in triplicate.
